# One-Year Mortality Rates Following Fragility Femoral Fractures in Patients Presenting to King Saud Medical City in Riyadh, Saudi Arabia: A Retrospective Study

**DOI:** 10.7759/cureus.28844

**Published:** 2022-09-06

**Authors:** Hamid T ALJohani, Ibrahim Alshugair, Shoog F Alfadhel, Elham A Alghamdi, Hussain Alkaff, Bander S Alrashedan, Hussain ALYousif

**Affiliations:** 1 Department of Orthopedic Surgery, King Saud Medical City, Riyadh, SAU; 2 Department of Orthopedic Surgery, King Fahad Medical City, Riyadh, SAU

**Keywords:** fracture, fragility fracture mortality, proximal femur fractures, fragility fractures, osteoporosis, geriatric trauma

## Abstract

Objective: The objective is to investigate one-year mortality rates following femur osteoporotic fractures, and to investigate factors that are associated with higher mortality rates.

Design: A retrospective study was conducted from 2010 to 2021 (11 years) of all patients who presented to King Saud Medical City, Saudi Arabia, and had a fragility fracture of the proximal or distal femur.

Patients: One hundred eight patients who sustained a proximal or distal femoral fracture, as a result of low-energy trauma, were included.

Results: The majority of our cohort (77.8%) had proximal femoral fractures, whereas only 22.2% had a distal femoral fracture. 55.6% were less than 75 years old, and 44.4% were 75 years or older. All patients had fallen from standing height. Hypertension and diabetes were the most common comorbidities among our cohort at 49.1% and 47.2%, respectively, but neither showed a statistically significant increase in the risk of mortality. When assessing the overall mortality, 21.3% of our patients had passed away. Although this finding was not statistically significant, mortality rates were found to be higher in patients with proximal femoral fractures compared to distal femoral fractures (25% vs. 8.3%, respectively, p=0.095). Patients with a normal bone mass density (BMD) had higher mortality rates as opposed to those with abnormal BMD (p=0.001).

Conclusions: Mortality rates are higher in proximal femoral fractures compared to distal femoral fractures. In addition, within our study cohort, patients with normal BMD had higher mortality rates. We recommend prospective studies that compare mortality rates between proximal and distal femoral fractures in patients with osteoporosis, as these studies would provide more accurate data. We also recommend having BMD measured in those patients to avoid further fractures in this patient population.

## Introduction

Osteoporotic fragility fractures can simply be defined as fractures that result from low mechanisms of injury, such as a fall from a standing height [1]. Proximal femoral osteoporotic fractures are very common and have high rates of morbidity and mortality, especially in the geriatric population [2]. Worldwide, osteoporotic fracture rates are increasing steadily due to the increase in life expectancy and increase in the aging population; they are projected to be three times higher by 2050, and hip fragility fractures are estimated to be around 6.26 million by then [3]. The mortality of proximal femoral fracture is reported to be 20%-30%, with recent strong evidence showing a significant decrease in mortality after the implementation of multidisciplinary approaches [4]. Several factors have been linked to increased risk of mortality after proximal femoral fractures, of which the timing prior to surgery and the presence of malignancy are the most significant. Other risk factors included residential status, presence of pulmonary disease, abnormal electrocardiogram findings, hypertension, and diabetes, as concluded by a large meta-analysis [5].

Distal femoral fragility fractures are another entity that has a mortality rate that is comparable to proximal femoral fractures. It accounts for approximately 4%-6% of femoral fractures, with one-year mortality rates of 30% [6]. As with proximal femoral fractures, mortalities are higher in patients with metastasized malignancies, renal failure, dementia, cardiac disease, or periprosthetic fractures [7].

Osteoporotic fractures increase the risk of subsequent fractures. In one study, the mortality of subsequent fractures was higher than that of the general population by three to four times, especially within one year; mortality declined over five years, but nonetheless remained higher than that of the general population [8]. In another study, men had higher mortalities when compared to women in both hip and non-hip fragility fractures; the absolute one-year mortality risk after a fragility fracture occurring at any site was 12.5% in women and 19.5% in men [9].

The aim of this current study is to compare one-year mortality rates between proximal (31-A extra-articular fracture, trochanteric area, 31-B extra-articular fracture, neck, 31-C articular fracture, head) and distal (33-A extra-articular fracture, 33-B partial articular fracture, 33-C complete articular fracture) femoral fragility fractures, and to determine the risk factors that increased mortality in both groups.

## Materials and methods

Study design

This is a retrospective cohort study that was conducted at King Saud Medical City (KSMC) (Level 1 Trauma Center) in Riyadh, Saudi Arabia. Electronic and paper records were searched for all patients who sustained osteoporotic proximal or distal femoral fractures due to low-energy trauma from 2010 to 2021. Patients who sustained proximal or distal femoral fractures after high-energy trauma or had pathological fractures secondary to bone lesions were excluded.

Our study included 108 patients. We collected the following data: age, gender, comorbidities, ambulatory status prior to presentation, mechanism of injury, presence of the previous fracture, aid used for mobilization after fracture management, and basic laboratory and radiological investigations at the time of admission (including blood pressure, x-ray, complete blood count, random blood sugar, and renal profile), follow-up duration, length of hospital stay, diagnosis of osteoporosis, presence or absence of complications, and mortalities, if present. Diagnosis of osteoporosis was done using the T-score from the lumbar spine (LS) and bilateral proximal femur (PF) on BMD (Horizon bone densitometry system, USA), using the Horizon software with the machine. (Horizon bone densitometry system, USA). The hospital follows the WHO criteria to diagnose osteoporosis. The criteria place the cut-off for osteopenia at a T-score between -1 and -2.5 and osteoporosis at a T-score below -2.5. BMD was done as part of postoperative woke up for the patients. Unfortunately, no pre-operative BMD was available for our patients in the institute. Thereafter, patients who were diagnosed with osteoporosis were referred to the endocrine department at the facility for medical management. Each patient’s current information and medical status were obtained from inpatient medical records, while incidences of death were obtained via telephone interviews with the patient's families.

Statistical analysis

Data analysis was performed using The Statistical Package for Social Sciences (SPSS) (Version 21.0, IBM Corp., Armonk, NY, USA) and Microsoft Excel to generate tables and charts. Descriptive statistics (mean, standard deviation, median, frequency, rates, minimum, and maximum) were used to describe the categorical study and outcome variables. Data were analyzed to measure the association between the categorical study and outcome variables. A p-value of <0.05 and 95% confidence intervals were used to report the statistical significance and precision of estimates. Independent t-tests were applied to the study variables.

## Results

Over the period from 2010 to 2021, 300 patients presented to KSMC with proximal or distal femoral fractures. All patients with high-energy femoral fractures, such as falls from significant height or road traffic accidents (RTA) were excluded. A total of 108 patients were included in the current study. Their data were analyzed. Males represented 45.5% of the study cohort. The age ranged from 63 to 80 years, with a median age of 73 years. The age was further categorized into two groups: 55.6% were less than 75 years old, and 44.4% were 75 years or older. The majority of the cohort had PF fractures (77.8%), and only 22.2% had distal femur fractures. Only 8.3% had prior fractures. Whether they were fragility fractures or fractures related to trauma was not indicated in our data. Regarding the mechanism of injury, all patients had fallen from standing height. Hypertension and diabetes were the most common comorbidities among the patients, with rates of 49.1% and 47.2%, respectively. The data relating to patient comorbidities are presented in Table 1.

**Table 1:** The distribution of comorbidities among our cohort DM - diabetes mellitus; CVD - cardiovascular disease

Comorbidities	Count	%
Hypertension	53	49.1%
DM	51	47.2%
CVD	14	13.0%
End-stage renal diseases	9	8.3%
Asthma	5	4.6%

Among the entire cohort, only 38.9% did not use any mobility aids. In addition, 29.6% used wheelchairs, 9.3% used walkers (Zimmer frames) with full weight-bearing as tolerated, 7.4% used crutches, and 14.8% used other mobility aids. Laboratory investigations done at the time of admission included random blood sugar and blood pressure. Random blood sugar ranged from 5.6 to 11.7 mmol/L, with a median random blood sugar of 7.2 mmol/L. Systolic blood pressure ranged from 130-155 mmHg, with a median systolic blood pressure of 140 mmHg (130-155). Diastolic blood pressure ranged from 69 to 85 mmHg, with a median diastolic blood pressure of 77 mmHg. Post-operative BMD revealed osteopenia in 12% of the cohort, osteoporosis in 42.6%, and normal BMD in 44.3%.

At the time of data collection, only 30.6% of the cohort were still actively following up in our hospital. The median length of the initial hospital stay ranged from, 6-16 days, with a median of 10 days. After searching the most recent follow-up data, 21.3% of our patients had passed away. Considering death as an outcome event in our study, a T-test was done to determine any significant correlation with the other variables. Among the risk factors studied in patients with normal BMD and with higher mortality rates, hypertension and diabetes mellitus were the most prevalent comorbidities present among our cohort; however, neither increased mortality rates, as seen in Table 2.

**Table 2:** The associations of final outcome (death, alive) with the independent variables HTN - hypertension, CVD - cardiovascular disease, ESRD - end-stage renal disease

		Final outcome				P-value
		Alive		Death		
		Count	%	Count	%	
Age	Less than 75	49	81.7%	11	18.3%	0.400
	75 and more	36	75%	12	25%	
Gender	Male	37	75.5%	12	24.5%	0.488
	Female	48	81.4%	11	18.6%	
Fracture location	Proximal femur	63	75.0%	21	25.0%	0.095
	Distal femur	22	91.7%	2	8.3%	
Previous fracture	Yes	6	66.7%	3	33.3%	0.398
	No	79	79.8%	20	20.2%	
HTN	Yes	47	88.7%	6	11.3%	0.018 (RR=0.37)
	No	38	69.1%	17	30.9%	
Diabetes mellitus	Yes	45	88.2%	6	11.8%	0.033 (RR=0.39)
	No	40	70.2%	17	29.8%	
CVD	Yes	12	85.7%	2	14.3%	0.730
	No	73	77.7%	21	22.3%	
ESRD	Yes	8	88.9%	1	11.1%	0.681
	No	77	77.8%	22	22.2%	
Asthma	Yes	4	80.0%	1	20.0%	>0.999
	No	81	78.6%	22	21.4%	
Other comorbidity	Yes	29	70.7%	12	29.3%	0.147
	No	56	83.6%	11	16.4%	
Bone mass density	Normal	29	61.7%	18	38.3%	0.001
	Osteopenia	12	92.3%	1	7.7%	
	Osteoporosis	42	91.3%	4	8.7%	

## Discussion

The aim of the present study was to compare the one-year mortality rates of patients who developed low-energy femoral fractures. Furthermore, we studied the factors that may influence the mortality rate according to the location of the femoral fractures. Proximal and distal femur fractures were associated with high mortality rates, especially among those who had chronic conditions or significant comorbidities. The findings from our study were measured retrospectively since their admission and up to one-year post-operatively for follow-up.

Our current study demonstrated that the majority of our patients had proximal femoral fractures (77.8%), whereas 22.2% had distal femoral fractures. The proximal femoral group was found to have a higher mortality rate. Furthermore, mortality was higher in males (24.5%) compared to females (18.6%). This contradicted a recent study done by Mubarak and associates in 2020. In their retrospective study, they included 189 patients, and mortality was assessed at different intervals of 30 days, 60 days, and one year. In contrast with our results, Mubarak et al. reported that compared to the mortality rate in the proximal femoral group (6.99% at 30 days, 14.52% at six months, and 21.51% at one year), the distal femoral group was greater at all times (9.68% at 30 days, 20.32% at six months, and 34.41% at one year). Moreover, the same study indicated that a higher number of patients with distal femoral fractures were treated without surgical intervention (15% vs. 4%) [10].

Fractures of any type at different sites of the body are considered risk factors for increased mortality rates and reduced survival, especially among osteoporotic patients. This was confirmed by a retrospective matched cohort study done by Brown and associates, who compared the survival between patients who suffered from fragility fractures and those who did not. Although the author only included patients aged 66 years and above, the results indicated a significant reduction in survival among those with fragility fractures. The authors concluded that fragility fractures were linked to decreased survival for up to six years after the fracture. The mortality risk doubled during the first year after fracture when deaths were seen in one in 11 females and one in seven males after a non-hip fracture and one in five females and one in three males after a hip fracture [9]. In another study that used data from the Korea Centers for Disease Control and Prevention’s National Health and Nutrition Examination Survey, the crude fatality rate was reported to be 14% for the 12-month period after a fracture. In the same study, different outcomes were measured including the prevalence of osteoporosis and fracture incidence. Adults above the age of 49 had osteopenia or osteoporosis at rates of 22.4% and 47.9%, respectively [11]. Another study also indicated that distal femoral fracture was associated with higher mortality rates, as determined by a retrospective analysis after adjusting for potential factors [12].

Other than the location of the femoral fracture, other factors have been linked to reduced mortality among osteoporotic femur fractures in patients aged 65 years or older. Aprato and associates studied mortality rates at different intervals, including six- and 12-month periods. Their study concluded that lower mortality rates were associated with early surgical intervention and early mobilization, specifically, walking ability [13].

The variability in mortality rates across the groups in our study can be explained by the differences in durations from presentation to surgery. In hip fractures, it is preferred to clerk the patient and start surgery within 36 hours of presentation [14]. In a study that compared hip-fracture patients who were admitted within 36 hours with those who underwent the surgery after more than 36 hours, the results indicated a lowered mortality rate among those who had the surgeries sooner [14]. Nonetheless, the aforementioned study concluded that the only medical condition that should be addressed prior to surgery is hyponatremia. Many factors have been identified to affect the waiting time for surgeries among patients with hip fractures. This was supported by a retrospective analysis done in Norway [15]; although the mean preoperative waiting time was 22.6 hours, delayed surgeries were associated with increased 30-day and one-year mortality rates [15].

Our current study has several limitations. First, it is a retrospective cohort study, which was conducted with data collected from prior documentation in patients’ files. Second, a large number of patient records have been excluded from this analysis due to missing data. Third, the study has a small sample size. However, it provided the baseline data for evaluating risk factors and measuring mortality rates after such injuries. Fourth, our population lacked pre-operative BMD. Fifth, many patients had lost follow-up for various reasons, which may have impacted the results. Finally, most of the cohorts’ x-rays were not clearly fit for AO classification correlation. In addition to that, the high proportion of patients with proximal femoral fractures may have influenced the mortality rates, which should be considered in future studies to allow the comparison of two equal samples.

## Conclusions

In this study, we investigated the one-year mortality rates following osteoporotic femoral fractures and the relevant factors that are associated with increased mortality rates. We conclude that mortality rates are higher in proximal femoral fractures compared to distal femoral fractures. In addition, within our study cohort, patients with normal BMD had higher mortality rates. Hypertension and diabetes mellitus were the most prevalent comorbidities among our sample; however, both did not show that they were risk factors for increased mortality rate. We recommend prospective studies that compare mortality rates between proximal and distal femoral fractures in patients with osteoporosis, as these studies would provide more accurate data. We also recommend having BMD measured in those patients to avoid further fractures in this patient population.
